# Optimization of Fibrin Scaffolds to Study Friction in Cultured Mesothelial Cells

**DOI:** 10.3390/ijms23094980

**Published:** 2022-04-29

**Authors:** Francesca Bodega, Chiara Sironi, Luciano Zocchi, Cristina Porta

**Affiliations:** Dipartimento di Fisiopatologia Medico-Chirurgica e dei Trapianti, Sezione di Fisiologia Umana, Università degli Studi di Milano, 20100 Milan, Italy; chiara.sironi@unimi.it (C.S.); luciano.zocchi@unimi.it (L.Z.); cristina.porta@unimi.it (C.P.)

**Keywords:** fibrinogen, fibrin gel, friction, mesothelial cells

## Abstract

To study the friction of cell monolayers avoiding damage due to stress concentration, cells can be cultured on fibrin gels, which have a structure and viscoelasticity similar to that of the extracellular matrix. In the present research, we studied different gel compositions and surface coatings in order to identify the best conditions to measure friction in vitro. We examined the adhesion and growth behavior of mesothelial cell line MET-5A on fibrin gels with different fibrinogen concentrations (15, 20, and 25 mg/mL) and with different adhesion coatings (5 μg/mL fibronectin, 10 μg/mL fibronectin, or 10 μg/mL fibronectin + 10 μg/mL collagen). We also investigated whether different substrates influenced the coefficient of friction and the ability of cells to stick to the gel during sliding. Finally, we studied the degradation rates of gels with and without cells. All substrates tested provided a suitable environment for the adherence and proliferation of mesothelial cells, and friction measurements did not cause significant cell damage or detachment. However, in gels with a lower fibrinogen concentration, cell viability was higher and cell detachment after friction measurement was lower. Fibrinolysis was negligible in all the substrates tested.

## 1. Introduction

Fibrin is a major component of intra- and extra-vascular blood clots that form at the sites of vessel wall damage, and also of the extracellular matrix. Commercially available fibrinogen and thrombin are combined to form a fibrin hydrogel that can be used as a scaffold for growing cells, for the regeneration of tissues, or to optimize cell adhesion, proliferation, and differentiation [1]. Fibrin has been extensively used as a biopolymer in tissue engineering due to a number of unique biological and physical characteristics, including its deformability and elasticity [2]. Moreover, fibrin facilitates cell adhesion, migration, and proliferation due to the presence of peptide chains and integrin binding sites on fibrinogen molecules [3]. Moreover, a fibrin glue can be produced by mixing thrombin and concentrated fibrinogen [4]. Varying the concentration of key components of fibrin gel, including fibrinogen and thrombin, leads to a change in the properties of fibrin scaffolds, affecting how cells proliferate, migrate, and differentiate within fibrin [5,6,7,8].

In this research, a fibrin gel was used to grow cells in order to measure the lubricating properties of cultured mesothelial cell monolayers. In our previous studies, we performed such measurements on ex vivo tissue samples [9,10,11], using the apparatus built to measure frictional force described by D’Angelo et al. [12]. This apparatus was adapted to perform measurements on cell monolayers [13]. When cells cultured on a hard substrate slide against each other under normal stress, they are easily destroyed due to stress concentration. In order to overcome this problem, cell monolayers can be cultured on a gel with a 3D network structure and a viscoelasticity similar to that of the extracellular matrix [14].

In the present research, we studied different gel compositions and different surface coatings in order to identify the best conditions to measure the friction coefficient of cell monolayers. For this purpose, we examined the adhesion and growth behavior of mesothelial cell line MET-5A on fibrin gels with different fibrinogen concentrations (15 mg/mL, 20 mg/mL, and 25 mg/mL) and with different adhesion coatings (5 μg/mL fibronectin, 10 μg/mL fibronectin, or 10 μg/mL fibronectin + 10 μg/mL collagen). We also investigated whether different concentrations of fibrinogen and different coatings influenced the coefficient of friction and the ability of cells to stick to the gel surface during sliding. Finally, we studied the degradation rates of gels with and without cells.

## 2. Results

**Figure 1:**
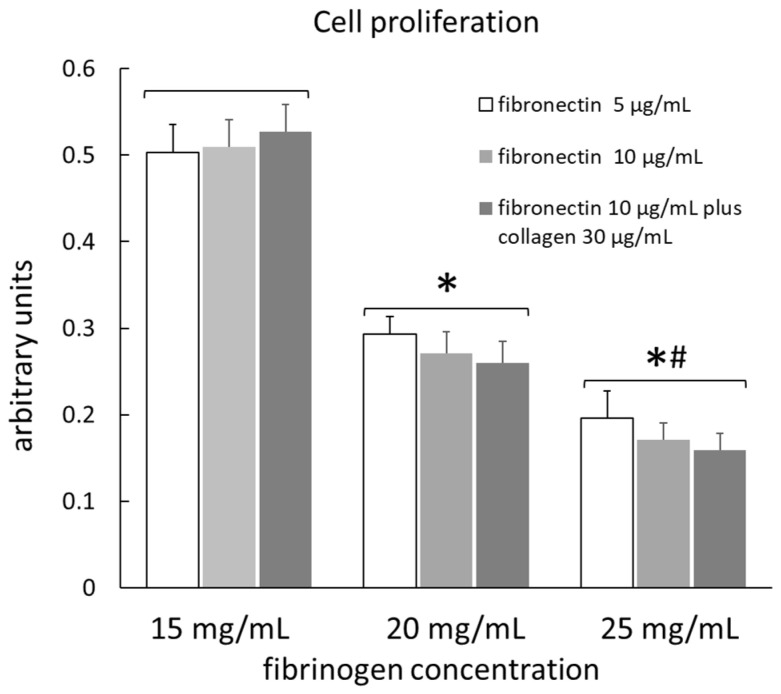
Cell proliferation assay. The viable cells seeded on different gels with different coatings were determined using a Cell Counting Kit (Cell Counting Kit-8, MCE, Monmouth Junction, NJ, USA). The results were obtained from 10 measurements for each type of substrate. *: significantly lower (*p* < 0.01) than gel with fibrinogen concentration of 15 mg/mL. #: significantly lower (*p* < 0.01) than gel with fibrinogen concentration of 20 mg/mL.

The number of viable cells seeded on different gels with different coatings, determined using a Cell Counting Kit (Cell Counting Kit-8, MCE, Monmouth Junction, NJ, USA), is shown in Figure 1. Data are means of 10 measurements for each type of substrate. The number of viable cells was not influenced by the different surface coatings in any type of gel, so the data from the three different coatings for each type of gel were grouped together. The number of viable cells on the gel with a fibrinogen concentration of 15 mg/mL (gel 1) was significantly greater than that of cells on the gel with a fibrinogen concentration of 20 mg/mL (gel 2); the latter, in turn, was significantly greater than that of cells on the gel with a fibrinogen concentration of 25 mg/mL (gel 3), indicating that the adhesion and proliferation of cells are facilitated in softer matrices.

**Figure 2:**
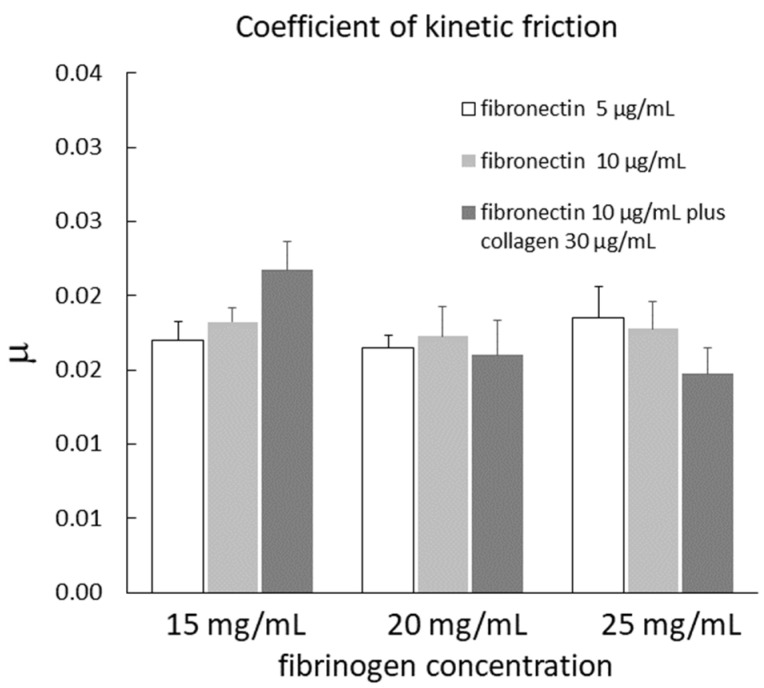
Coefficient of kinetic friction (µ) of cells seeded on different gels with different coatings. Data are means of four measurements for each type of substrate.

The coefficient of kinetic friction (µ) of cells seeded on different gels with different coatings is shown in Figure 2. The data are means of four measurements for each type of substrate. The coefficient of kinetic friction of the cells was not influenced by the different surface coatings in any type of gel. After grouping the data from the three different coatings for each type of gel, µ was 0.019 ± 0.001, 0.016 ± 0.001, and 0.017 ± 0.001 in gel 1, gel 2, and gel 3, respectively. These values were not significantly different from each other, indicating that the type of gel did not influence µ.

**Figure 3:**
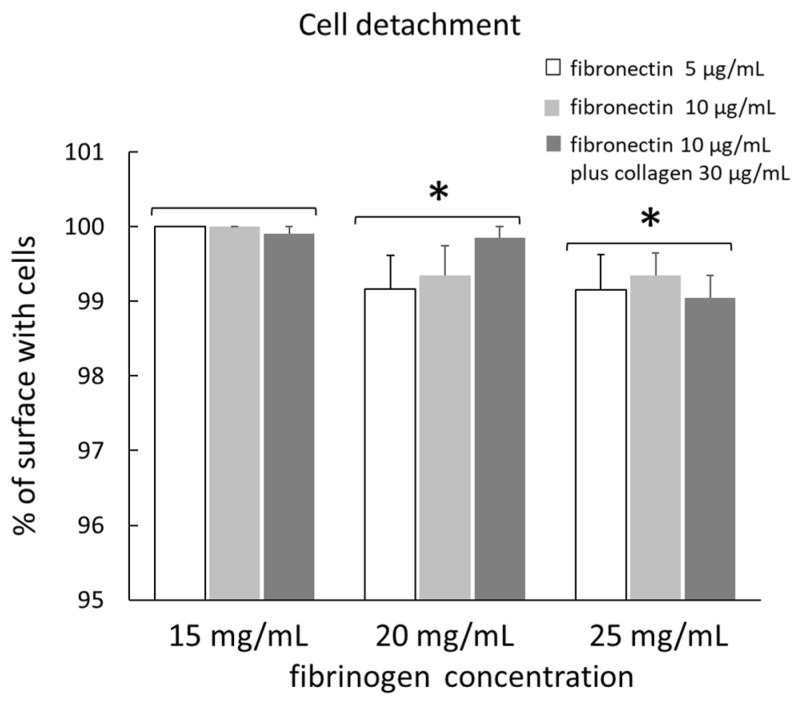
Percentage of gel surface with cells, relative to the total surface, in different gels with different coatings, after the measurement of µ. Data are means of 4 measurements for each type of substrate. *: significantly lower (*p* < 0.01) than in gel with fibrinogen concentration of 15 mg/mL.

The detachment of cells seeded on different gels with different coatings after µ measurement is shown in Figure 3. The data are means of four measurements for each type of substrate. The percentage of surface with cells, relative to the total surface, was not influenced by the different surface coatings in any type of gel. However, in gel 2, this value tended to increase in fibronectin 10 µg/mL plus collagen 30 µg/mL as coating, although this difference was not significant. After grouping the data from the three different coatings for each type of gel, the percentage of surface with cells was 99.97 ± 0.03, 99.45 ± 0.21, and 99.18 ± 0.19 in gel 1, gel 2, and gel 3, respectively. In gel 2 and gel 3, the percentage of surface with cells was significantly lower than in gel 1, but this value was not significantly different between gel 2 and gel 3.

**Figure 4:**
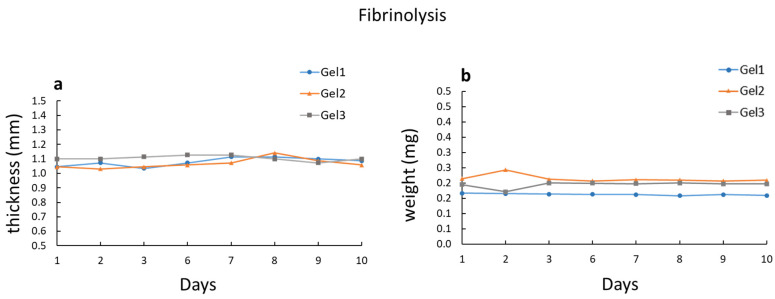
(**a**) Degradation of gel with cells. Gel degradation was estimated in the 3 types of gel by measuring gel thickness for 10 days after seeding cells. (**b**) Degradation of gel without cells, kept at 37 °C in culture medium. Gel degradation was estimated in the 3 types of gel by measuring gel weight for 10 days. Gel 1 = 15 mg/mL fibrinogen; Gel 2 = 20 mg/mL fibrinogen; Gel 3 = 25 mg/mL fibrinogen.

The degradation of gels with cells is shown in Figure 4a. Gel degradation was estimated by measuring the gel thickness for 10 days after seeding cells. Gel thickness remained essentially unchanged throughout the 10 days, for all gel types. Gel degradation was also measured in gels without cells, but kept at 37 °C in culture medium. In this case, gel degradation was estimated by measuring the gel weight for 10 days. Also in this case (Figure 4b), the gel weight remained essentially unchanged throughout the 10 days, for all gel types. Therefore, gel degradation was negligible for a relatively long time, which was sufficient for µ measurements.

**Figure 5:**
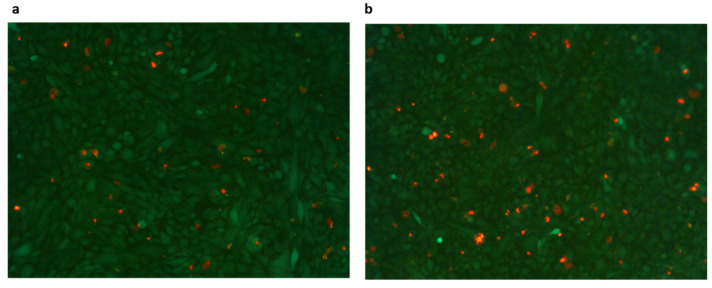
Representative image of gel before (**a**) and after (**b**) µ measurement. Cells were marked with Live and Dead Cell Assay (ab115347, Abcam, Cambridge, UK), which labels live cells in green and dead cells in red.

Figure 5 shows a representative image of gels before and after µ measurement. Cells were marked with Live and Dead Cell Assay (ab115347, Abcam, Cambridge, UK), which labels live cells in green and dead cells in red. Dead cells did not seem to increase in number after µ measurement.

## 3. Discussion

In this research, fibrin gels were developed as a substrate to grow mesothelial cells in order to determine their lubricating properties after adapting an apparatus previously used to measure the frictional force on ex vivo tissue samples [9,10,11]. When we tried to perform these measurements on cells cultured on a Petri dish, the monolayer was easily destroyed, probably due to stress concentration. Pitenis et al. [15] studied the friction coefficient of human corneal epithelial cells cultured on glass, and found that no measurable cell death occurred after 10,000 cycles (approximately 24 h) at average contact pressures of 1 kPa. However, in these experiments, cells slid on a soft hydrogel biomaterial. In order to reduce cell detachment, cell monolayers can be cultured on a gel that has a 3D network structure and a viscoelasticity similar to that of the extracellular matrix [14]. To our knowledge, the only research in which cells were cultured on a gel in order to measure friction is that of Chen et al. [14]. They studied the sliding friction of HUVEC monolayers cultured on PNaSS gel. We also tried to use this substrate, but unfortunately, the mesothelial cells were not able to attach and proliferate on this gel. Then, we tested a more natural substrate, a fibrin gel, prepared from fibrinogen and thrombin. In the present research, we studied fibrin gels with different fibrinogen concentrations and different surface coatings in order to identify the best conditions to measure the coefficient of friction in mesothelial cell line MET-5A in vitro.

A number of variables can influence the structure of a fibrin gel, including the local pH, ionic strength, and calcium concentrations [16]. However, the most important variables are the fibrinogen and thrombin concentrations. A higher fibrinogen concentration produces gels with thicker fibers and smaller pores [17], while increasing the thrombin concentration produces gels with thinner, more tightly packed fibrin fibers [16]. In this research, in order to reduce the variables, we chose to keep a fixed thrombin concentration and only varied the fibrinogen concentration. When fibrin gels have been used as scaffolds for culturing cells in previous studies, very different concentrations have been used, ranging from 4 mg/mL [6] to 75 mg/mL [18]. The fibrinogen concentrations that we chose were 15, 20, and 25 mg/mL. In fact, concentrations lower than 15 mg/mL produce very soft gels, which are difficult to handle, while concentrations higher than 25 mg/mL are very difficult to solubilize. An important feature of the gels used in this research is transparency, which allows the cells to be inspected routinely with an optical microscope.

The coefficient of kinetic friction (µ) measured on the cell line was not influenced by the type of coating or by the type of gel. These results suggest that the surface properties of mesothelial cells were not influenced by the substrate on which they grew and that the mechanical proprieties of the gel did not influence µ. The mean value of µ was 0.0175 ± 0.001. This value was similar to those measured on pleural tissue specimens (approximately 0.025) [19], and on mesothelial cells in vitro (0.021 ± 0.001 and 0.032 ± 0.002 in 4/4 RM-4 and CARM-L1 TG3 cell line, respectively) [13]. Chen et al. [14] found that the surface sliding friction of endothelial cell monolayers cultured on soft PNaSS gel was much greater than that exerted on the walls of blood vessels in vivo. They explained this result mainly using two reasons: (1) the friction was tested under a normal pressure 10 to 100 times higher than the pressure exerted on the endothelial cells by the red-cell membrane in vivo, and (2) because of the poor growth of the glycocalyx layer on the mesothelial cells cultured in vitro. Negative pressure in the pleural space holds the opposed mesothelial surfaces together, so that they are actually pushing one against the other [20]. The spatially averaged pressures acting on the opposed mesothelial surfaces during resting or moderately increased ventilation should not exceed 10 cmH_2_O [21]. In our study, the effect of this pressure was reproduced by applying a normal force to the tissue ranging from ~0.5 to ~8 g, corresponding to a pressure on the mesothelium from ~0.8 to ~12.9 cm H_2_O, similar to that estimated in vivo [10]. Our results suggest that the surface properties of mesothelial cells in culture are similar to those of mesothelial surfaces in situ. However, it should be emphasized that the previous measurements on the tissue were performed ex vivo, and sample removal and storage procedures may have partially damaged the glycocalyx, therefore altering the surface properties.

The count of cells seeded on different gels with different coatings, determined by a Cell Counting Kit (Cell Counting Kit-8, MCE), showed that the adhesion and proliferation of cells were not influenced by the different surface coatings, while they were facilitated in softer matrices. This finding was also confirmed by the data on cell detachment. In fact, after µ measurement, the percentage of surface with cells relative to the total surface was significantly higher in gels with the lowest fibrinogen concentration (15 mg/mL). This value was not significantly different between 25 mg/mL gel and 20 mg/mL gel, although it was slightly higher in the latter. The percentage of cells that remained attached to the gel was over 99% in all gel types, indicating a very low detachment of cells during µ measurement. Chen et al. [14] found that the percentage of HUVECs that remained attached to the gel surface after the friction test gradually decreased with testing time, being only 40% after 1800 s. Moreover, the morphology of most of the HUVECs changed, indicating that the HUVECs were deformed by the frictional shear. In our test, the morphology of cells was not altered, as shown in Figure 5. However, our test did not last more than 300 s and the applied pressure was also lower. Additionally, Dunn et al. [22], who studied the friction coefficient between soft hydrogel biomaterial and live human corneal epithelial cells cultured on glass, found that the detachment of the cells was directly correlated with the sliding cycles run.

Quantitative analysis of gel degradation showed that in all types of tested gels, fibrinolysis was very low, both in gels with cells and in those without cells. Willerth et al. [6] tested the degradation rate of fibrin gels used for embryonic stem cell proliferation and differentiation. They found that the degradation rate depended on the fibrinogen concentration. With the highest concentration used (12 mg/mL), after 3 days, gels with cells retained around 80% of the fibrinogen initially present, while gels without cells retained around 95%. On the other hand, gel degradation was not influenced by the thrombin concentration. Similarly, Lee et al. [17] found that the dissolution rate of fibrin gels used for the delivery of bioactive vectors was significantly lower at higher fibrinogen concentrations. They found that with the higher concentration used (36 mg/mL), after 10 days, gels without cells kept in PBS at 37 °C lost around 20% of their weight. Additionally, Ahmed et al. [23] observed the rapid degradation of fibrin hydrogels used for articular cartilage tissue engineering, after the encapsulation of C5.18 cells.

Aprotinin, a known serine protease inhibitor, can be used to prevent the breakdown of fibrin [17,24]. In a previous study, in which we measured the µ of mesothelial cells cultured on fibrin gel [13], we added aprotinin to the medium. Nevertheless, the present findings indicated that, since, for friction measurements, gels are generally used within 4–7 days, the addition of aprotinin to the medium is not necessary. We do not know why fibrinolysis was negligible in our specimens. It could be due to the different compositions of the gel or the low content of plasmin in the fibrinogen used.

In summary, fibrin gels can be used as substrates to grow cells in order to measure their lubricating properties. All the substrates tested provided a suitable environment for the adherence and proliferation of mesothelial cells, and friction measurements did not cause significant cell damage or detachment. However, in gels with lower fibrinogen concentrations, the number of viable cells was higher, and cell detachment after friction measurements was lower. Finally, fibrinolysis was negligible in all substrates tested.

## 4. Materials and Methods

### 4.1. Fibrin Gel Preparation

Fibrin gels were formed by mixing fibrinogen and thrombin solutions. Gels were prepared on 25 mm Petri dishes and on a 60 mm Plexiglas plate, from which, once the gel had solidified, 4 disks with a radius of 1 cm and a thickness of around 1 mm were punched out by a holepunch and placed in Petri dishes.

The three gels tested were prepared maintaining the final concentration of thrombin at a constant (3 U/mL), and varying the final concentrations of fibrinogen (sc-473503, Santa Cruz, Dallas, TX, USA) to 15 mg/mL (gel 1), 20 mg/mL (gel 2), and 25 mg/mL (gel 3). Fibrinogen was dissolved by laying it on warm (37 °C) NaCl 1.1% under gentle agitation. Then, 20× thrombin solution was prepared by dissolving 60 U/mL of thrombin (T4648, Merck KGaA, Darmstadt, Germany) and 20 mM CaCl_2_ in NaCl 1.1%. Fibrinogen solution was poured on the Petri dishes or the Plexiglas plate (950 µL or 2850 µL, respectively), and a volume of 50 or 150 μL of 20× thrombin was added. To mix the solutions, the Petri dishes and the plate were gently rotated. Gels were allowed to polymerize for 1–2 h at room temperature, and then the 4 disks were punched out and placed in Petri dishes. These gels can be stored at 4 °C for 24 h. Before cell plating, gels were coated with three different surface coatings: bovine fibronectin (F1141, Merck KGaA, Darmstadt, Germany) 5 µg/mL, bovine fibronectin 10 µg/mL, or bovine fibronectin 10 µg/mL plus 30 µg/mL bovine collagen type I (M12SA, Cell Guidance Systems LLC, St. Louis, MO, USA) dissolved in culture medium. Petri dishes were rinsed with coating solutions and allowed to dry under UV light for 1 h.

### 4.2. Cell Culture

The MeT-5A cell line, derived from pleural fluids obtained from non-cancerous human individuals, was purchased from the European Collection of Cell Cultures (ECACC) and cultured in RPMI 1640 medium (R0883, Sigma) plus 15% fetal bovine serum (ECS0180L, Euroclone, Milan, Italy), 100×U/mL penicillin plus 100 mg/mL streptomycin (ECB3001D, Euroclone, Milan, Italy), 2 mM L-glutamine (ECB3000D, Euroclone, Milan, Italy), and 25 mM HEPES (ECM0180D, Euroclone, Milan, Italy).

Cultures were kept in a 5% CO_2_–95% air humidified incubator (Napco, model 5415, Thermo Fisher Scientific, Waltham, MA, USA) at 37 °C, fed every 2–3 days, and routinely subcultured with 0.05% trypsin–2mM EDTA solution (ECM0920D, Euroclone, Milan, Italy).

### 4.3. Cell Seeding on Gels

Gels were sterilized by 1 h of UV exposure. Then, cells were seeded on the gel surface at a density of 2–4 × 10^4^ cells/cm^2^. A confluent monolayer of cells was generally reached 2 days after plating, as assessed by inspection through an inverted microscope (Axiovert 100, Zeiss, Oberkochen, Germany). The morphology of the cells cultured on gel was not different from that of the cells cultured on plastic culture flask.

### 4.4. Cell Proliferation Assay

To study cell adhesion and proliferation on different substrates, the viable cells were determined by a Cell Counting Kit (Cell Counting Kit-8, MCE, Monmouth Junction, NJ, USA). This kit uses a highly water-soluble tetrazolium salt, WST-8 (2-(2-methoxy-4-nitrophenyl)-3-(4-nitrophenyl)-5-(2,4-disulfophenyl)-2H-tetrazolium, monosodium salt), to count living cells. WST-8 is reduced by dehydrogenases in cells to generate an orange-colored product (formazan), which is soluble in the tissue culture medium. The amount of the formazan dye generated by dehydrogenases in cells is directly proportional to the number of living cells.

To perform the assay, different gels with different coatings were formed on 25-well plates. The same number (4 × 10^4^ cells) of cells was seeded on each well, and after 2 days, 10 μL of CCK-8 solution was added to each well of the plate. The plate was incubated for 4 h at 37 °C and 5% CO_2_ atmosphere. Then, from each well, 100 mL samples were transferred to a 96 micro-plate wells. The absorbance of the content of each well was measured using a spectrophotometric plate reader (Infinite M^®^, Tecan, Männedorf, Switzerland) at 450 nm.

### 4.5. Friction Measurements

**Figure 6:**
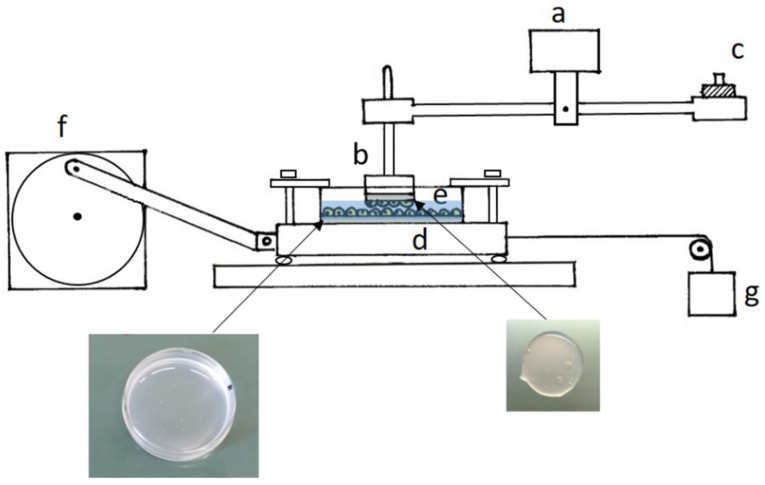
Schematic drawing of the device used to measure frictional forces: (**a**) force transducer; (**b**) Plexiglas rod on which a gel disk with a mesothelial monolayer was glued; (**c**) counterweight; (**d**) sliding platform; (**e**) Petri dish containing cells seeded on gel; (**f**) electric motor; (**g**) differential transformer.

The apparatus used to measure the frictional force was that described by D’Angelo et al. (2004) [12]. It consists of a sliding platform connected through inextensible threads to the core of a differential transformer (Lynearsyn 565 DT, Sanborn, Waltham, MA, USA), and a balance arm held stationary at its fulcrum by a force transducer (Figure 6). Specimens to be tested were fixed to the sliding platform, which was driven sinusoidally over a distance of 1 cm by an electric motor, and to a Plexiglas rod attached to one end of the balance arm, which could rotate to maintain contact between the sliding surfaces. The balance arm was held horizontally, and counterweights added to its other end enabled us to change the normal force applied to the tissue from ~0.5 to ~8 g, corresponding to a pressure on the mesothelium from ~0.8 to ~12.9 cmH_2_O. The Petri dish containing cells seeded on the gel was fixed to the sliding platform, while a gel disk with a mesothelial monolayer on one surface was fixed with cyanoacrylate glue to the Plexiglas rod attached to the balance arm. The frictional force on the direction of motion was measured by the force transducer. Under any given condition, the coefficient of kinetic friction (μ) was computed as the slope of the relationship between the load and the frictional force.

Measurements of μ were made at room temperature (21–28 °C) in phosphate-buffered solution (PBS; in mmol/L: NaH_2_PO_4_ 19.2, Na_2_HPO_4_ 80.7, NaCl 38.6).

After each friction measurement, cells were observed through an inverted microscope to check if sliding caused cells to detach from the gel. To quantify cell detachment, the Petri dishes were observed through a grid, which divided the surface into 36 parts. For each part, the percentage of surface without cells was evaluated. Taking into account the surface of each part with respect to the total, the percentage of the total surface without cells was then obtained.

### 4.6. Fibrinolysis

Although fibrinolysis is a useful phenomenon in some fibrin gel applications, in our case, it was not desirable, as it would lead to the destruction of the substrate on which the cells lay. Therefore, it was an important process to consider. Fibrinolysis was evaluated on both gels on which cells had been seeded, and on gels without cells, because the cells could contribute in some way to the degradation. In both cases, gels were maintained in culture medium at 37 °C and 5% CO_2_ atmosphere.

To evaluate gel degradation in gels with cells, the thickness of the gel was measured by observing the gel with an inverted microscope and determining the number of graduation marks by which the fine adjustment was turned in order to shift the focus from the lower to the upper surface of the gel. The lower surface was marked with an ink mark on the bottom of the Petri dish, while the upper surface was identified by the presence of the cells.

To evaluate the degradation of gels without cells, gels were weighed each day and the weight difference was calculated. Their initial swollen mass (in mg) was taken after allowing the gels to swell for 1 h in culture medium. The samples were then placed in an incubator and weighed every day, after they were gently blotted to remove excess medium before weighing.

### 4.7. Cell Labeling

In some specimens, before and after friction measurements, cells were marked with Live and Dead Cell Assay (ab115347, Abcam, Cambridge, UK), which allows for the differential fluorescent labeling of live and dead cells. The dye was diluted 20× in culture medium, and cells were incubated at 37 °C for 1 h before imaging.

### 4.8. Statistics

The results are presented as mean ± S.E. Linear regression between frictional force and load was computed with the least-squares method. Statistical assessment was conducted using variance analysis. The level of significance was taken as *p* ≤ 0.01.

## Data Availability

Not applicable.
